# Response to: Comment on “Optic Nerve Sheath Diameter Ultrasound Evaluation in Intensive Care Unit: Possible Role and Clinical Aspects in Neurological Critical Patients' Daily Monitoring”

**DOI:** 10.1155/2018/4631581

**Published:** 2018-11-01

**Authors:** M. Toscano, G. Spadetta, P. Pulitano, M. Rocco, V. Di Piero, O. Mecarelli, E. Vicenzini

**Affiliations:** ^1^Department of Human Neurosciences, Sapienza University of Rome, Italy; ^2^Organs Harvest Center coordinator, Sapienza University of Rome, Italy; ^3^Department of Medical and Surgical Science and Translational Medicine, Sapienza University of Rome, Italy

We thank the authors for the interesting comments on our paper [1] and for truly appreciating our work as well.

We absolutely agree that optical nerve sheath diameter (ONSD) ultrasound (US) measurement could be influenced from own personal experience and skills. In general, this represent a general criticism towards US imaging per se, since scanning protocols are sometimes not performed properly and require long training to acquire the experience. However, this represents an adequate price to be paid for precious real-time noninvasive data to be obtained, a price otherwise minimized when US are properly performed, as in this case (see the methods section) [2], thus emphasizing the capability of US to depict real-time imaging and haemodynamics. This is particularly crucial in intensive care units settings, where emergencies sometimes require quick and rapid decisions.

In our cases we performed imaging directly at the bedside in the ICU, with a general machine that was at disposal for multiple investigations, with a specific setting of power reduction for the eye. Measurements were calculated directly on the apparatus, however, without the possibility of exporting the DICOM files. Images were then printed in Sony B/W paper and thereafter digitized. Thus, even though the achieved data came from an exact “computerized” measurement, images rescanned on the PC from analogical printing (actually of low quality, we apologize for this) were used for the presentation. Markers were recoloured on the original ones to magnify them and to make it more understandable even by the reader “nonexpert” in the field. This may be the reason for the actual low-quality image presentation.

Regarding the “30° test”, although we do not have direct experience with this technique, we think that this is a further confirmation of the high potential of ONSD measurement. Thus, further studies with multispecialist cooperation will certainly add more valuable proof of ONSD's usefulness in several clinical conditions.

Concerning the comment on the experience needed for the ONSD US measurements to be performed, it is now us who are in disagreement with the authors. Though orbital and eye ultrasound is a very specific section for the ophthalmologist, the specific measurement of the ONSD may be learned without such difficulties with a short training, even by those who are not completely involved in the US world. Specialized medical personnel, such as anaesthesiologists, learn even more complicated tasks, as well as the nurses in intensive care units who are often very skilled in technical capabilities. For this reason, the take-home message we wanted to give with our paper [2] is that in the ICU the evaluation of ONSD with US should be more diffuse in daily monitoring, because it may be important to noninvasively identify those patients that are developing fatal raised intracranial pressure (ICP). And this could—and should—be done also by specifically trained caring physicians, without awaiting and calling for the consultant and thus avoiding any further increase in the time required for the raised ICP to be promptly detected.

## References

[1] De Bernardo M., Rosa N. (2018). Comment on “Optic nerve sheath diameter ultrasound evaluation in intensive care unit: possible role and clinical aspects in neurological critical patients’ daily monitoring”. *BioMed Research International*.

[2] Toscano M., Spadetta G., Pulitano P. (2017). Optic nerve sheath diameter ultrasound evaluation in intensive care unit: possible role and clinical aspects in neurological critical patients’ daily monitoring. *BioMed Research International*.

